# Bovine Leukemia Virus Infection in Neonatal Calves. Risk Factors and Control Measures

**DOI:** 10.3389/fvets.2018.00267

**Published:** 2018-10-25

**Authors:** Vanesa Ruiz, Natalia Gabriela Porta, Marina Lomónaco, Karina Trono, Irene Alvarez

**Affiliations:** ^1^Instituto Nacional de Tecnología Agropecuaria—Instituto de Virología, Centro de Investigaciones en Ciencias Veterinarias y Agronómicas, Buenos Aires, Argentina; ^2^Consejo Nacional de Investigaciones Científicas y Técnicas, Buenos Aires, Argentina

**Keywords:** bovine leukemia virus, neonatal calves, colostrum, milk, dairy farms, proviral load

## Abstract

Bovine leukemia virus (BLV) is the causative agent of enzootic bovine leukosis (EBL). Although efficient eradication programs have been successfully implemented in most European countries and Oceania, BLV infection rates are still high worldwide. BLV naturally infects cattle, inducing a persistent infection with diverse clinical outcomes. The virus infects lymphocytes and integrates a DNA intermediate as a provirus into the genome of the cells. Therefore, exposure to biological fluids contaminated with infected lymphocytes potentially spreads the virus. Vertical transmission may occur *in utero* or during delivery, and about 10% of calves born to BLV-infected dams are already infected at birth. Most frequently, transmission from dams to their offspring occurs through the ingestion of infected colostrum or milk. Therefore, although EBL is not a disease specific to the neonatal period, during this period the calves are at special risk of becoming infected, especially in dairy farms, where they ingest colostrum and/or raw milk either naturally or artificially. Calves infected during the first week of life could play an active role in early propagation of BLV to susceptible animals. This review discusses the main factors that contribute to neonatal BLV infection in dairy herds, as well as different approaches and management practices that could be implemented to reduce the risk of BLV transmission during this period, aiming to decrease BLV infection in dairy herds.

## Enzootic bovine leukosis

Enzootic bovine leukosis (EBL), also called bovine lymphosarcoma or bovine leukemia, is an infectious disease naturally occurring in cattle. The viral etiology of this lymphoproliferative disease was first described in 1969, when Miller and colleagues discovered that lymphocytes of cows with persistent lymphocytosis produced viral particles that were visible by electron microscopy after *in vitro* culture (1). The etiological agent of EBL is bovine leukemia virus (BLV), an oncogenic retrovirus member of the genus *Deltaretrovirus* (family *Retroviridae)*. This genus also includes the Human T-lymphotropic viruses (HTLV) 1, 2, and 3, and the Simian T-lymphotropic viruses (STLV) 1, 2, and 3 (2). Once BLV infects a cell, it integrates a DNA intermediate as a provirus, both randomly and permanently, into the genome of the host cell. This virus preferentially infects B cells (3). In cattle, the polyclonal expansion of B cells, which is characteristic of animals with persistent lymphocytosis, occurs almost exclusively within the CD5+ IgM+ B cell subset (4, 5) and these B cells are also the primary target for BLV proviral integration (5, 6). However, other cell types like CD4^+^ T cells, CD8^+^ T cells, γ/δ T cells, monocytes and granulocytes can also be infected by BLV (3, 7–10). The free virus is rarely detected *in vivo*, and it is believed that it appears only during the acute stage of infection, before the development of neutralizing antibodies (11).

Infection with deltaretroviruses is characterized by a long incubation period and a variety of clinical outcomes. Almost 70% of BLV-infected animals are asymptomatic carriers of the virus and are referred to as aleukemic. Since these animals do not present clinical and/or hematological manifestations of the disease, they can be identified only by the presence either of specific antibodies or of integrated viral DNA (provirus) (12). Approximately 30% of infected cattle develop a non-malignant proliferation of untransformed B-lymphocytes, termed persistent lymphocytosis, a condition characterized by an increase in the number of peripheral blood circulating B-lymphocytes (above 10,000/mm^3^) (13). During persistent lymphocytosis, animals may suffer from immunological dysregulation, as evidenced by opportunistic infections (e.g., mastitis) (14–17). Less than 5% of BLV-infected animals develop malignant B-cell lymphosarcoma, which occurs between 1 and 8 years after infection. This local proliferation of B cells can occur within different organs and tissues like the spleen, liver, heart, abomasum, uterus, lymph nodes, and spinal cord (12, 13, 18, 19). Although clinical signs associated with lymphosarcoma are highly variable, as they depend on the affected organ(s), these tumors may lead to a series of defects that are finally incompatible with the survival of the animals.

Although cattle are the predominant natural hosts for BLV infection, water buffalo, zebu, yak and alpaca can also become naturally infected (20–23). Although experimental infection has been studied in goats (24), rabbits (25), rats (26, 27), and chickens (28), the most consistent model to study BLV infection seems to be sheep. In this model, the complete onset of the disease occurs in a relatively short period of time (approximately 18 months) and the frequency of leukemia/lymphoma is high (close to 100%) (29, 30).

BLV infection is widely disseminated throughout the world and is listed by the World Organization for Animal Health (OIE) as a disease of importance to international trade (31). Several countries have established eradication programs and control measures based on “test and eliminate” and “test and segregate” approaches, that have been very successful in Western Europe (32–34), New Zealand (35), and Western Australia (36). However, in several nations of Eastern Europe, including Ukraine, Bulgaria and Croatia, the disease is still present. In addition, high levels of BLV prevalence are still documented in North and South America, and some Middle Eastern and Southeast Asian nations. The updated information on the incidence and distribution of EBL worldwide has been recently reviewed by Polat et al. (37). Argentina, where the disease is endemic, is the South American country with the highest BLV prevalence, with an individual prevalence of 80% in dairy farms of the main productive areas of the country (38).

Different techniques have been developed to diagnose BLV infection. These can be assigned into two main groups: those consisting of antibody-based serological tests and those detecting the proviral genome by PCR. Antibody-based serological tests, which include agar gel immunodiffusion (AGID) and the enzyme linked immunosorbent assay (ELISA), are the most common and reliable way to diagnose BLV infection. AGID is recognized as the official import/export test (31), but is less sensitive than ELISA, which is commonly used for routine diagnosis. Antibody-based serological tests can use different types of antibodies such as antibodies against the viral envelope glycoprotein (gp51) or antibodies against the core polypeptide (p24). Generally, the former has higher titer and appear earlier than the latter. As mentioned above, BLV infection can also be diagnosed by techniques that detect the proviral genome. These techniques include standard PCR, nested PCR or real-time quantitative PCR (qPCR) (39–43). The advantages of the PCR-based methods are that they allow detecting BLV infection several weeks before it is possible to detect antibodies and that they allow differentiating positive from negative calves in the presence of maternal antibodies.

## Transmission

BLV is transmitted mostly through the transfer of infected cells. Biological fluids like blood, colostrum, and milk are potential sources for the transmission of BLV (44–46), and their infectivity depends on the lymphocyte count of the fluid (46).

Horizontal transmission of BLV occurs mainly associated with iatrogenic procedures, such as dehorning, tattooing, vaccination, castration, and rectal tact (46). The implementation of management practices to minimize exposure of the animals to BLV has proved to reduce overall herd prevalence in some studies (33, 47, 48). However, dairy producers in Argentina that have applied corrective measures to prevent the iatrogenic blood contact have not been able to reduce the BLV prevalence rate in the herd, which suggests that other ways of transmission are playing a key role under natural conditions (49). Transmission by biting insects such as Tabanid flies has also been documented (50), but its relative importance under natural conditions is uncertain. Vertical transmission includes perinatal and postnatal infection. Perinatal infection may occur either *in utero* or during delivery. *In utero* infections under field conditions have been demonstrated by testing newborn calves before colostrum feeding, and proved to be between 4 and 18% (51–55). This natural *in utero* BLV infection has been found to be independent of the breed (52, 53), dam age, dam parity, and time of BLV infection in the dam (42), but has been associated with maternal lymphocytosis (54, 55), malignant lymphoma (54), and maternal viral loads (52). Experimental infection of cows during pregnancy has also been found to result in seropositive calves at birth, indicating that calves had been infected *in utero* (56). Recently, Sajiki et al. (57) reported the direct evidence of intrauterine infection in two pregnant dams with a high proviral load (PVL). These authors detected BLV DNA in both of the newborns delivered via cesarean section by nested PCR, and found that the amplified BLV-*env* gene sequences from the dams and the newborns were completely identical. These authors also detected BLV provirus in placental and cord blood, but not in amniotic fluid, suggesting that placental and cord blood might be routes of vertical BLV transmission. (52) investigated the frequency of perinatal BLV infection in field conditions in Japan and observed that 10 out of 129 (7.7%) calves born from BLV-infected cows were infected in the birth canal, and 14 (10.8%) were infected *in utero*. In addition, they found no correlation between the rate of birth canal infections and the assistance during parturition or the number of births per dam (52).

Postnatal vertical infection from dams to calves occurs through consumption of infected colostrum or milk. Nowadays, in most dairy farms, calves are fed with colostrum and milk during the first 60 days of life. Colostrum can come directly from their dams or be administered by nipple bottle or oroesophageal tubing. During this period, calves are also fed with bulk-tank milk and balanced feed and then they are moved to pastures occupied exclusively by cattle of similar age and weight. Several authors have described the presence of provirus and infectious virus both in milk and colostrum from most BLV-infected cows and in bulk tank milk (58–60). Thus, both milk and colostrum are sources of infection to neonatal calves. However, milk and colostrum can also contain BLV specific antibodies (58, 59). Therefore, the potential protective or infective role of colostrum and milk in natural transmission of BLV is still not clear and has been the subject of multiple studies that have suggested contrasting roles.

The protective role of colostrum has been reported by different groups. Van der Maaten et al., for example, performed an experimental study with newborn calves to which they gave 10^6^ to 10^9^ BLV-infected lymphocytes in either colostrum free of BLV specific antibodies or colostrum from BLV-infected cows. Their results revealed that BLV antibody-containing colostrum protected calves from infection (61). Lassauzet et al. found similar results in a 3-year prospective study in a dairy herd where calves were housed in individual hutches. They observed that the number of calves that had not received BLV colostral antibodies and had become infected was significantly higher than that of calves that had received BLV colostral antibodies (62). Similarly, Nagy and colleagues reported that the incidence of BLV infection was higher in colostrum-deprived calves and suggested that the administration of BLV-positive colostrum reduces the risk of infection compared with colostrum deprivation (63).

The infectivity of colostrum and milk has been experimentally demonstrated in sheep inoculated or oral administered with these secretions, and subsequently examined for the development of infection (64, 65). Milk-borne transmission has also been reported in calves born to BLV-negative dams that had been fed milk from BLV-infected cows (66), as well as in calves fed on colostrum and milk from their BLV-positive dams since birth and then reared in complete or partial isolation from infected cattle (45). Another study performed in dairy herds showed that almost all new BLV infections occurred in calves born to uninfected dams. However, when feeding with bulk milk was stopped and calves began to be fed only milk from BLV-free cows, no new infections occurred in the following 2.5 years (67).

Different studies performed by our group in dairy herds of Argentina have revealed that the presence of provirus in colostrum is significantly correlated with the blood PVL (60, 68). Our studies have also shown that the colostrum of individual cows shows different provirus/antibody profiles, and that consumption of colostrum with infected cells and a poor content of antibodies could play a critical role in BLV propagation during young age (59, 60). In addition, when analyzing the relationship between the level of BLV antibodies and the PVL in blood and milk of lactating cows under natural conditions, we found a negative correlation, suggesting that the consumption of raw milk containing provirus or free virus particles and low levels of antibodies could favor BLV transmission to calves (58). All these findings suggest that the feeding management of young calves could greatly influence the risk of milk-borne transmission.

## Importance of BLV infection during the neonatal period

Although EBL is not considered an infectious disease causing abortions or neonatal mortality, special attention should be given to neonatal calves in dairy herds, especially in those with high prevalence of BLV infection. Epidemiological studies carried out by our group in dairy herds in Argentina have shown that calves infected during the first week of life play an active role in early BLV propagation to non-infective calves, since their PVL increases during the first 12 months and remains high for years (68). Since high levels of BLV infection are associated with higher probability of transmission (69), the presence of animals with high PVL is epidemiologically dangerous. This could be a reason for the high incidence observed in Argentinean dairy herds before first parturition, when approximately half of pregnant heifers are already infected, and between 20 and 44% of them have high PVL (60, 70). Culling the animals with high PVL might be a strategy for reducing overall incidence in the herds, but this would be economically feasible only in herds with low prevalence of high PVL cows.

Peak rates tend to occur in the interval in which heifers are being bred, calving, and entering the milking herd, a time of intensive human intervention, closer physical contact and exposure to older cows with higher BLV infection rates (46, 49). However, it has also been reported that BLV infection can occur during the first 2 years of life, when young animals are still not in contact with adult cows. At this stage, the only potential sources of virus are their own mothers, bulk tank milk, and calves that were born infected (8–11% in dairy herds of Argentina) (60, 70).

## Preventive strategies to reduce perinatal transmission

Considering all the above, it is clear that management strategies focused on reducing BLV transmission during the perinatal period could be of great help to diminish the prevalence of infection in dairy herds. Different strategies should be implemented at specific times from parturition.

## Preventive strategies during parturition

As previously mentioned, the frequency of *in utero* transmission is significantly correlated with the maternal viral load. Therefore, selecting breeding cows according to their viral loads could reduce the number of intrauterine infections. In addition, considering that BLV can also be transmitted through the birth canal, cesarean section in dams with high PVL should be aseptically conducted to minimize the risk of BLV transmission to newborn calves.

Ideally, heifers should be separated from adult cows with high rates of infection before the calving process and newborn calves should be removed from their dams at birth and placed in a clean dry area to be fed good-quality colostrum during their first 12 h of life. Additionally, calves born infected should be identified as soon as possible and segregated from the herd. Some of these management practices are included in control programmes based on “test and segregate,” and have been useful to decrease prevalence or even achieve eradication of the disease (33, 47, 71).

## Preventive strategies when feeding calves with colostrum and milk

Colostrum is the main source of nutrients and maternal immunoglobulins for the newborn calf. The timely feeding of high-quality and adequate volumes of uncontaminated colostrum is a key factor, essential to the health and survival of neonatal dairy calves (72). Since the incidence of BLV infection in dairy herds is usually high, natural suckling from dams should be avoided and replaced by artificial feeding with either a high-quality colostrum bank or colostrum replacer. The high-quality colostrum bank could be obtained as pooled colostrum from BLV-negative dams. However, in dairy herds with high rates of infection, this would be almost impossible to conceive. In this case, a colostrum bank should be created from dams with high levels of BLV-specific antibodies (59). Alternatively, the colostrum bank could be pre-treated to render it non-infectious. Kanno et al. reported that a useful means of inactivating the infectivity of BLV is by freezing the colostrum. Thus, these authors used frozen-thawed or untreated colostrum from a BLV-infected cow, and then isolated the leukocytes from these colostra to use them to inoculate sheep. The sheep inoculated intraperitoneally with the leukocytes from the frozen-thawed colostrum remained BLV-negative until 9 weeks after inoculation, whereas the sheep inoculated with the cells of the untreated colostrum became infected with BLV at 3 weeks after inoculation (73). However, a disadvantage of using frozen colostrum is that it requires large amounts of refrigerated storage space and considerable time for thawing and warming prior to feeding.

Currently there are several commercially available colostrum supplements or replacers made from dried bovine colostrum. Colostrum may be dried by means of different methods such as freeze-drying, microwave vacuum evaporation, and spray-drying. Comparison of these methods has shown that spray-drying is the most cost-effective and allows obtaining a dried colostrum in which immunoglobulin quantity and function are preserved (74). Moreover, our group has recently reported that the experimental spray-drying process is effective in inactivating infectious BLV in colostrum. In that study, we inoculated susceptible lambs with treated (spry-dried) or untreated colostrum spiked with BLV-infected cells and found that lambs that had received the untreated colostrum showed evidence of infection 60 days post-inoculation (75).

Pasteurization of milk is another feasible strategy to ensure the inactivation of BLV without significantly altering the concentration of immunoglobulins or their physiological activities (75–77). It has been reported that sheep inoculated with milk experimentally contaminated with BLV and treated by a simulated high-temperature short-time (HT ST) pasteurization procedure do not become infected or develop tumors (75, 77). In addition, HT ST pasteurization has the additional benefit of helping to limit the spread of Johne's disease and other pathogens (78). All these findings suggest that performing pre-treatments to both colostrum and bulk tank milk before they are administered to calves might help to prevent early BLV infection.

## Preventive strategies in raising calves

In most dairy herds, raising calves are generally fed with bulk tank milk (with approximately 10–15% of their body weight daily), for 60 days. Considering a dairy herd with high prevalence of BLV infection, calves might be receiving a constant supply of virus during that period. Supplementation of the milk with BLV-specific antibodies able to neutralize the virus prior to intake, which would complement the neutralizing capacity provided by natural antibodies already present, could be an interesting approach. In this regard, a promising and practical strategy that has been explored since the early 1990s is the supplementation of the milk diet of calves with specific antibodies from egg yolk (IgY) (79–81). This IgY is deposited in egg yolk in large quantities, making chickens an ideal source of specific polyclonal antibodies (82). The supplementation of newborn calves' diets with egg yolk powder enriched in specific IgY antibodies has been successfully used in preventive and therapeutic treatments against bovine rotavirus diarrhea (79, 80, 83, 84). Thus, our group is currently evaluating the use of specific IgY antibodies as a milk supplement to achieve passive protection of newborn calves against BLV in field conditions. For this purpose, non-infected newborn calves (tested negative for BLV by nested PCR) are being fed with non-pasteurized bulk tank milk, supplemented with egg powder enriched with BLV-specific IgY during their first 60 days of life. This study would help to know the real risk of milk-borne transmission and the potential use of IgY technology to prevent this route of infection.

## Final conclusions

The greatest advance in dairy health in the last years has been the shift to disease prevention, rather than treatment (85). In Argentina and many other countries worldwide, BLV infection is endemic, with high prevalence in dairy farms. Thus, several control measures should be implemented to control the dissemination of the disease until a treatment or vaccine become available. Experimental evidence has indicated that BLV infection can easily propagate during the perinatal period when young animals are still not in contact with infected adult cows. Therefore, different approaches should be simultaneously implemented to effectively interrupt BLV transmission to calves and finally have herds with decreased levels of BLV infection.

## Author contributions

VR and IA conceived the idea of the review. All authors wrote sections of the manuscript. The submitted version of the manuscript was read, edited, and approved by all authors.

### Conflict of interest statement

The authors declare that the research was conducted in the absence of any commercial or financial relationships that could be construed as a potential conflict of interest. The handling Editor declared a shared affiliation with the authors.
